# Synthesis and Structural Characterization of SiO_2_ Nanoparticles Using Extract of *Gracilaria Crassa* Via Green Chemistry Approach

**DOI:** 10.1002/open.202400356

**Published:** 2024-11-21

**Authors:** Vanathi Palanimuthu, Rajiv Periakaruppan, Valentin Romanovski, Ayyarappan Bharathi, Karungan Selvaraj Vijai Selvaraj, Somasundaram Anukeerthana, Rajendran Nishanthi, Gurumoorthy Vanajadevi

**Affiliations:** ^1^ Department of Biotechnology Sri Ramakrishna College of Arts & Science Coimbatore 641006, Tamil Nadu India; ^2^ Department of Biotechnology PSG College of Arts & Science Coimbatore 641014, Tamil Nadu India; ^3^ Department of Materials Science and Engineering University of Virginia Charlottesville VA 22904 USA; ^4^ Department of Genetics and Plant Breeding Agricultural College and Research Institute, Vazhavachanur 606753 Thiruvannamalai India; ^5^ Vegetable Research Station Tamil Nadu Agricultural University, Palur 607102 Cuddalore India

**Keywords:** SiO_2_ nanoparticles, *Gracilara crassa*, Antioxidant, Characterization

## Abstract

Biologically synthesized nanoparticles via biological entities are produced with less negative impact on the environment and without expensive chemicals, which have biocompatibility and are eco‐friendly. *Gracilaria crassa* is a well‐known marine red alga that is edible and unique and is the commercial source of agar and agarose production. In this work, *Gracilaria crassa*‐mediated SiO_2_ NPs were synthesized by the green synthesis method. In order to characterize the physicochemical properties of the synthesized SiO_2_ NPs, a variety of microscopic and spectroscopic approaches were used, including imaging with Field emission scanning electron microscopy, Energy‐dispersive X‐ray analysis, UV spectrophotometry, X‐ray diffraction analysis, Fourier‐transform infrared spectroscopy, Zeta potential, and Thermo gravimetric analysis. These results shown that *Gracilaria crassa*‐mediated SiO_2_ NPs were amorphous nature, negatively charged −15.5 mV and spherical shape in size of 20–50 nm. The antioxidant capacity of synthesised SiO_2_ NPs was examined by 2,2‐Diphenyl‐1‐picrylhydrazyl assay and it observed as IC_50_ value of 49.4 μg/mL denotes that could counteract the production of free radicals and oxidative stress. The characteristics of the synthesized *Gracilara crassa*‐mediated SiO_2_ NPs suggest their application as potential antioxidant agents.

## Graphical abstract

This study demonstrates the eco‐friendly synthesis of SiO_2_ nanoparticles using *Gracilaria crassa*, revealing their amorphous, negatively charged, and spherical nature. These nanoparticles exhibit significant antioxidant potential, highlighting their promise as sustainable nanomaterials with potential therapeutic applications.

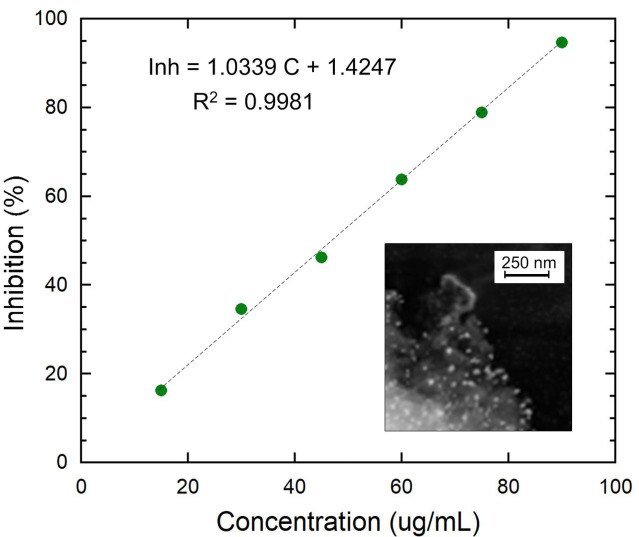

## 1 Introduction

Nanotechnology is the most auspicious technology of the 21st century. Nanoparticles can display an extensive range of size‐dependent properties. Within the size range, the gap between the small molecules and bulk materials the nanoparticles use as linking in state of energy. Generally, nanoparticles are classified based on their morphology, composition, dimensionality, agglomeration, and uniformity.^[1]^

Silicon dioxide (SiO_2_) nanoparticles are highly versatile materials used across various industries due to their unique physical and chemical properties, including high surface area, tunable porosity, and biocompatibility.[^2^, ^3^] In biomedical applications, these nanoparticles are employed as carriers for drug delivery, where their porous structure allows for high drug loading capacity and controlled release.^[4]^ Functionalization with specific molecules enables targeted delivery, enhancing therapeutic efficacy while minimizing side effects. Additionally, SiO_2_ nanoparticles are used in bioimaging and diagnostics by being doped with fluorescent dyes or contrast agents, providing stability and biocompatibility suitable for long‐term imaging.^[5]^ They are also incorporated into biosensors for the detection of biomolecules, where their large surface area facilitates the attachment of enzymes, antibodies, or DNA, increasing sensor sensitivity.^[6]^ In environmental applications, SiO_2_ nanoparticles play a crucial role in water treatment by adsorbing heavy metals,^[7]^ organic pollutants,^[8]^ and microorganisms,^[9]^ and are incorporated into membranes for improved filtration efficiency.^[10]^ They are also used in air purification systems to capture fine particulate matter and pollutants,^[11]^ and in soil remediation to immobilize heavy metals and other contaminants,^[12]^ reducing their environmental impact. Industrially, silica nanoparticles serve as catalysts or catalyst supports in chemical reactions,^[13]^ particularly in the petrochemical industry, where their high surface area enhances reaction rates and selectivity. They are added to coatings,^[14]^ paints,^[14]^ and varnishes to improve mechanical properties, scratch resistance, hydrophobicity, and UV protection, extending the lifespan of coated surfaces. In the construction industry, SiO_2_ nanoparticles enhance the mechanical properties and durability of cement and concrete by reducing porosity, increasing compressive strength, and improving resistance to chemical attacks.^[15]^ In the energy sector, these nanoparticles are being explored for use in lithium‐ion batteries, where they can serve as anodes or be incorporated into electrolytes to improve ionic conductivity and thermal stability.^[16]^ They also contribute to more efficient solar cells by enhancing light absorption and reducing reflectivity,^[17]^ and in fuel cells,^[18]^ they are used as catalyst supports or membrane reinforcements to improve performance and durability. In cosmetics and personal care products, SiO_2_ nanoparticles are utilized in sunscreens for their UV‐blocking properties, offering transparency on the skin without leaving a white residue.^[19]^ They are also added to skincare products to improve texture, absorb excess oil, and deliver active ingredients more effectively. In the food and agriculture sector [20], silica nanoparticles are used in food packaging materials to enhance barrier properties and extend the shelf life of perishable goods.^[21]^ In agriculture, they improve soil quality, enhance nutrient delivery, and protect plants from pests and diseases.[^22^, ^23^] Overall, SiO_2_ nanoparticles offer a wide range of applications, from advanced medical treatments to sustainable environmental solutions, with ongoing development promising even more innovative uses in the future.

The nanoparticles synthesized using the green chemistry approach has been environmentally friendly and cost‐effective which is an alternative to physical and chemical methods.^[24]^ The biosynthesized nanoparticles have an effective control over the new drug discoveries.^[25]^ Biologically synthesized via biological entities such as bacteria, algae, fungi, yeast, and plants are produced without toxicity and expensive chemicals which has the lack of toxic and eco‐friendly. Using biological entities as a green synthesis approach has the potential to produce nanomaterials and is widely used in agriculture, as targeted drug delivery and has biochemical applications.[^26^, ^27^] The SiO_2_ NPs can be synthesized by various physical, chemical, and biological methods. The green synthesis methods for SiO_2_ NPs have several benefits and can also help to mitigate some of the shortcomings of the other synthesis methods that were previously stated.^[28]^ The benefit of using the green synthesis of SiO_2_ NPs is that it is a cost‐effective, time‐efficient, and sustainable technique.^[29]^ SiO_2_ NPs have a wide range of applications in many different technical fields, thus their demand will only grow in the future. An emerging trend in the “green synthesis” of nanoparticles is the use of algae species, due to its simple to work, and use also capable of absorbing and accumulating inorganic metallic ions. And using algae to create nanoparticles is a quick, natural, and environmentally benign process and inexpensive and non‐toxic technique.[^30^, ^31^] *Gracilaria crassa* is a well‐known marine red alga that is edible and unique to India. It is a member of the *Gracilariaceae* class.^[32]^ *Gracilaria* is the commercial source of agar and agarose production. *Gracilaria crassa* contains a good quantity of essential amino acid, carbohydrates; macro and micro nutritional elements and low lipid content were reported.^[33]^ Nowadays, due to the high pharmaceutical demand, antioxidant is very much the focus of research. Many researches have been conducted on antioxidant chemicals and red macroalgae, which are abundant, easy to culture, and comprise significant natural resources.^[34]^

The potential of the edible sea algae *Gracilaria crassa* in the production of SiO_2_ NPs hasn't been investigated, though. This investigation aims to study SiO_2_ NPs synthesized using *Gracilaria crassa* by green chemistry approach. The synthesized nanoparticles were characterized by using various analytical techniques. For further studies, the antioxidant activity of SiO_2_ NPs was carried out.

## 2 Materials and Methods

### 2.1 Materials and Reagents

The marine red algal species *Gracilaria crassa* and Tetraethyl orthosilicate (TEOS) were obtained from the R.K Algae project centre located in Ramanathapuram District Mandabam Northeast (PALKBAY) sea area and Sri Mahalakshmi Scientific Company in Coimbatore respectively.

### 2.2 Preparation of Algal Extract

The seaweed was washed and rinsed with water to remove dirt and mud. 5 g of seaweed was weighed and chopped into small pieces. It was then ground in mortar and pestle finely. The grounded algae were added to 100 mL of deionized water and heated until the solution was reduced to half. The algal extract can be obtained by filtering the heated solution using the Whatman No 1 filter paper. The extracted seaweed solution was stored in cold condition which can be used for synthesis of nanoparticles.

### 2.3 Synthesis of SiO_2_ NPs

For the synthesis of SiO_2_ NPs 10 mL aqueous extract of algae was heated at 80 °C using a hot plate. The mixture containing 5 mL TEOS, 3 mL distilled water, and 7 mL Ethanol was placed in a magnetic stirrer at 80 °C for 10 mins. The seaweed extract of 10 mL was added to the heated TEOS mixture. Then this solution was placed in a magnetic stirrer at 80 °C for 30 mins. This solution was cooled and transferred to the sterile petri dish. The solution was dried into powder by placing it in a hot air oven. The obtained powder was collected and stored for characterization studies.

### 2.4 Characterization of SiO_2_ Nanoparticles

The SiO_2_ NPs were characterized by several techniques. The optical property of Si NPs was analyzed by using UV‐Visible spectrophotometer (Shimadzu/206–26300–48 model). The wavelength range of 220–800 nm was chosen for UV‐Visible spectrophotometry because it covers both the ultraviolet (UV) and visible regions of the electromagnetic spectrum, which are essential for analyzing the optical properties of SiO₂ nanoparticles. The UV region (220–400 nm) is particularly important for detecting electronic transitions in nanoparticles, as SiO₂ nanoparticles often exhibit absorbance in this range due to interactions between light and their electronic structure. The visible region (400–800 nm), although SiO₂ nanoparticles are not inherently visible, is useful for confirming transparency and ensuring that no significant absorbance occurs, which could indicate the presence of impurities or particle aggregation. Therefore, the 220–800 nm range provides a comprehensive understanding of the nanoparticles’ optical properties, ensuring that they are properly characterized in terms of size, stability, and purity. X‐ray diffraction (XRD) analysis was determined the crystalline nature of the synthesized SiO_2_ NPs (X'Pert Pro Panalytical). Fourier‐transform infrared spectroscopy (FTIR) analysis was performed to identification and characterization of a functional group and molecules in synthesized nanoparticles (SHIMADZU Miracle 10 Model). The SiO_2_ NPs were observed for their size and morphology using Field Emission Scanning Electron Microscope (FE‐SEM) and EDAX analysis of SNPs to determine morphology and the atomic weight percentage of the elements (TESCAN‐MIRA3 XMU). For thermal characterization Thermo Gravimetric Analyzer (TGA) used (EXSTAR/6300 model).

### 2.5 Antioxidant Activity

The antioxidant potential of SiO_2_ nanoparticles synthesized from *Gracilaria crassa* algae extract was investigated using the 2,2‐Diphenyl‐1‐picrylhydrazyl (DPPH) assay, method that assesses free radical scavenging activity by measuring the absorbance values of sample solutions at various concentrations. UV‐vis absorbance was recorded at 520 nm using a microplate reader against DMSO. The reaction mixture comprised 1 mL of 0.1 mM DPPH solution combined with varying concentrations (15, 30, 45, 60, 75, and 90 μg/mL) of synthesized SiO_2_ NPs. Upon interaction with substances like SiO_2_ NPs capable of donating hydrogen atoms, the initially violet DPPH solution turns yellow, indicating reduction. The following formula was used to determine the antioxidant for Radical Scavenging Activity (%RSA).

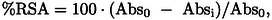




where Abs_0_ – absorption of Control; Abs_i_ ‐ absorption of sample.

## 3 Results and Discussion

### 3.1 SiO_2_ NPs Characterization

**Figure 1:**
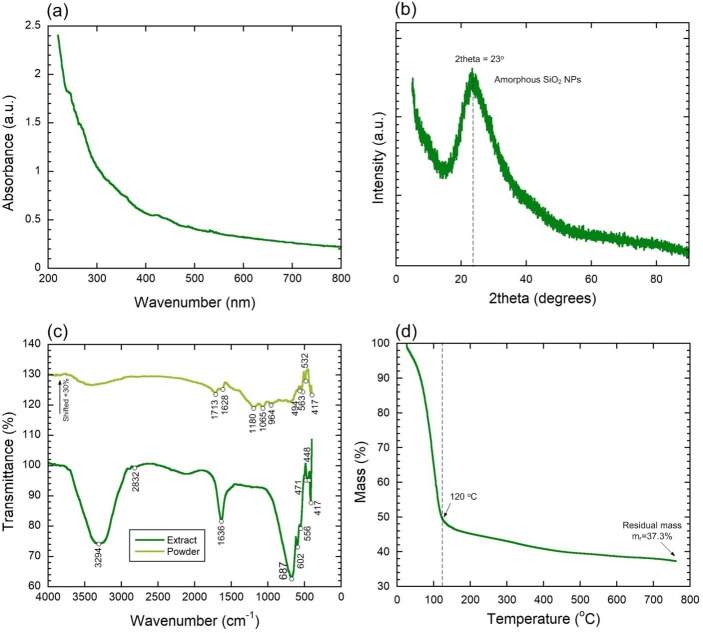
UV‐vis spectra (a), XRD diffractogram (b), FTIR spectra (c), and TGA (d) analysis of synthesized SiO_2_ NPs.

The ultraviolet‐visible analysis was used to determine the optical properties of the SiO_2_ NPs prepared from TEOS. The study examined the effect of SiO_2_ NPs size on electronic absorption and behaviour. A graph was plotted using the wavelengths and optical densities of UV‐Visible spectra readings for the synthesis of SiO_2_ NPs from *Gracilaria crassa*. The optical absorption of specific light wavelengths can be measured using the photometric measurement technique. This technique was performed using a UV‐visible spectrophotometer in the wavelength range of 220–800 nm,^[35]^ as shown in Figure 1a. The UV‐visible spectrophotometer in the wavelength range of 250 nm is likely used to analyse the absorbance and transparency of the SiO_2_ NPs. This range covers the ultraviolet (UV) and visible light spectra, allowing to assess the material's optical properties and potentially gain insights into its composition, size, and surface characteristics.

XRD analysis was performed for synthesized SiO_2_ NPs, and the typical diffraction patterns are shown in Figure 1b. The pattern displays a broad peak characteristic of the amorphous phase of SiO_2_ NPs[^36^, ^37^] at angle of 2θ=23° (JCPDS No., 00–001‐0649) confirming its amorphous nature.^[38]^ No any of other peaks were detected.

The Fourier transform infrared (FTIR) is a useful tool for determining and characterising the molecules and functional groups (chemical bonds) that are present in a compound [36]. The seaweed extract and SiO_2_ NPs were applied to the quartz slide, and an FTIR Spectrometer (Shimadzu IR Prestige‐21 model) was the utilized to capture the 4000–400 cm^−1^ range of materials′ FTIR spectra. The FTIR spectrum of SiO_2_ NPs produces a peak at 1713, 1628, 1180, 1065, 964, 563, 532, 494, 417 cm^−1^. The O−H stretching vibration can be seen in the absorption band region at 3487 cm^−1^. The O−H bending can be seen at 1628 cm^−1^. Asymmetric Si‐O−Si stretching were observed at 1064.71 cm^−1[38]^ as shown in Figure 1c. The FTIR spectra of the seaweed extract produces an absorption peak at 3294, 2832, 1635, 687, 602, 556, 471, 447, 417 cm^−1^ as shown in figure 1c. The peak observed at 1636 and 3294 cm^−1^ were organic nitrates and hydroxy group respectively,^[39]^ Figure 1c.

A Thermo gravimetric Analyzer (TGA) was utilized to analyse the thermal stability and mass loss of the SiO_2_ NPs.^[38]^ The temperature ranges up to 105 °C is usually associated with water loss [40, 41] (Figure 1d). The rest of this mass loss region up to 120 °C can be related to the decomposition of C−N bonds.[^42^, ^43^] The next mass loss range (>120 °C) of synthesized SiO_2_ NPs associated with the loss of organic matter (functional groups found by FTIR).

**Figure 2:**
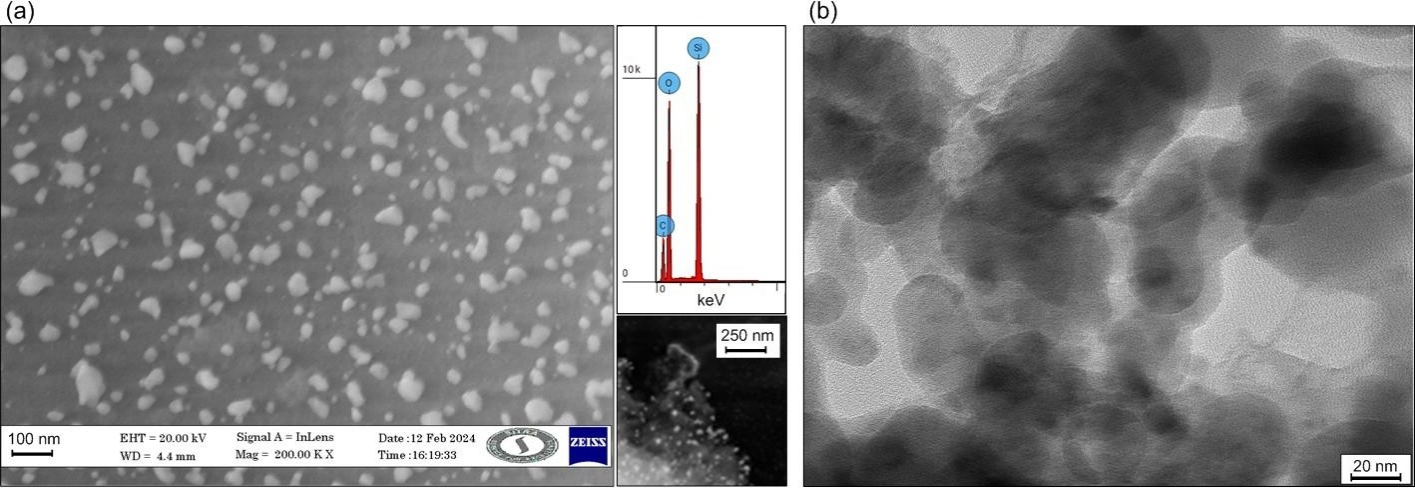
SEM‐EDS (a), and TEM images (b) of synthesized SiO_2_ NPs.

Field emission scanning electronic microscope was used to find out the morphologies of the *Gracilaria crassa* mediated SiO_2_ NPs. The magnification of FESEM images showed the spherical and amorphous shown in Figure 2a. The typical size of synthesized nanoparticles was in the range 20–50 nm (Figure 2b) and their shape was similar mentioned in the.^[44]^ The TEM image also confirmed the amorphous phase of synthesized SiO_2_ NPs. Likewise, Ganesan et al., 2023^[35]^ green synthesis the SiO_2_ NPs by *Halymenia floresia* aqueous extract has the spherical in shape.

The Energy‐dispersive X‐ray analysis was conducted with SEM analysis to expose the composition and purity of the synthesized SiO_2_ NPs. The EDAX spectra show the silicon and oxygen atomic percentages as 25.76 and 74.24 respectively and the weight percentages of silicon and oxygen were 62.15 and 37.85 respectively. This can confirm the presents of both SiO_2_ and functional groups.^[45]^

**Figure 3:**
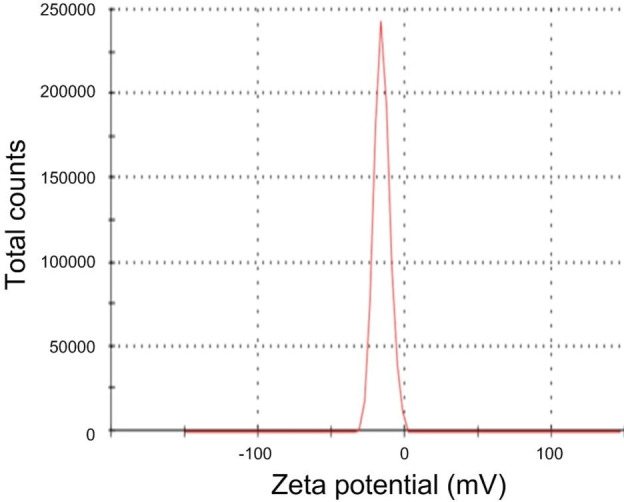
Zeta potential analysis of SiO_2_ NPs.

Zeta potential analysis is parameter used to characterize the electrophoretic mobility and electrostatic charge on the surface of nanoparticles which are expressed in millivolts (mV). The synthesized nanoparticles from the *Gracilara crassa* were zeta potential performed. The value of the SiO_2_ NPs was −15.5 mV has the negative charge thus the particles are stable as shown in Figure 3. Marousek et al., reported that synthesized SiO_2_ NPs assess the zeta potential value as −20.3 mV are highly stable and negative charge.^[46]^ This is crucial for understanding the colloidal stability of the nanoparticles in various applications.

### 3.2 Antioxidant Analysis

**Figure 4:**
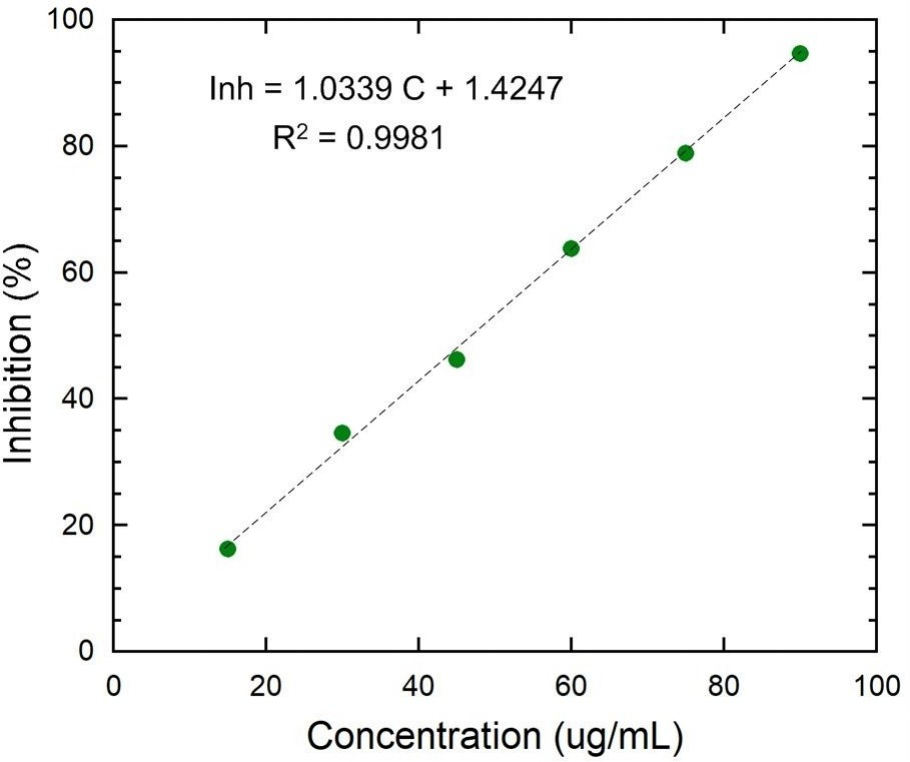
Antioxidant analysis of SiO_2_ NPs.

The maximum antioxidant activity was found at 90 μg/mL of SiO_2_ NPs and lowest activity was found at 15 μg/mL of SiO_2_ NPs (Figure 4). IC50 value was found to be 49.4 μg/mL. The SiO_2_ NPs may interact with free radical and reduce the oxidative stress. The coefficient of determination of obtained model was 0.9981, which is an indicator of a high degree of approximation of the model by experimental data and indicates a strong positive relationship close to functional.

## 4 Conclusions

The green procedure for synthesis of the SiO_2_ NPs was done in this current study using *Gracilara crassa* aqueous extract. The synthesized nanoparticles were characterized by various methods. The absorbance peak was found at 250 nm in UV‐Vis spectra primarily characterized and confirmed the nanoparticles. In microscopic technique FESEM showed the images of nanoparticles morphologic, spherical and in size of 20–50 nm. XRD characterization discovered the amorphous nature of the synthesized SiO_2_ NPs. The antioxidant activity of green synthesised SiO_2_ NPs revealed that in enables the scavenging ability for the free radicals and oxidative stress. Synthesised SiO_2_ NPs might be beneficial for the development of novel and more potent antioxidants agent. Consequently, using the green approach in manufacturing nanoparticles is more economical and commercial approach also toxic free and bio compactable. *Gracilara crassa* mediated SiO_2_ NPs has offering antioxidant potential which paved way for further studies in biomedical application.

## Author Contributions

**Vanathi Palanimuthu**: investigation, formal analysis, validation, data curation, writing‐original draft; **Rajiv Periakaruppan**: conceptualization, supervision, investigation, formal analysis, validation, data curation, writing‐original draft, writing‐review and editing, project administration; **Valentin Romanovski**: investigation, formal analysis, validation, data curation, writing‐original draft, writing‐review and editing; **Ayyarappan Bharathi**: formal analysis, validation, data curation, investigation. **Karungan Selvaraj Vijai Selvaraj**: formal analysis, validation, data curation, investigation; **Somasundaram Anukeerthana**: formal analysis, validation, data curation, investigation; **Rajendran Nishanthi**: formal analysis, validation, data curation, investigation; **Gurumoorthy Vanajadevi**: formal analysis, validation, data curation, investigation.

## Conflict of Interests

The authors declare no competing interest with any previous work.

5

## Data Availability

All data, models, and code generated or used during the study appear in the submitted article.
